# One-Year Old Dormant, “Non-culturable” *Mycobacterium tuberculosis* Preserves Significantly Diverse Protein Profile

**DOI:** 10.3389/fcimb.2020.00026

**Published:** 2020-01-31

**Authors:** Kseniya A. Trutneva, Margarita O. Shleeva, Galina R. Demina, Galina N. Vostroknutova, Arseny S. Kaprelyans

**Affiliations:** Federal Research Centre “Fundamentals of Biotechnology” of the Russian Academy of Sciences, A.N. Bach Institute of Biochemistry, Moscow, Russia

**Keywords:** dormant cells, non-culturable cells, *Mycobacterium tuberculosis*, 2D electrophoresis, proteomic profile

## Abstract

For adaptation to stressful conditions, *Mycobacterium tuberculosis* (*Mtb*) is prone to transit to a dormant, non-replicative state, which is believed to be the basis of the latent form of tuberculosis infection. Dormant bacteria persist in the host for a long period without multiplication, cannot be detected from biological samples by microbiological methods, however, their “non-culturable” state is reversible. Mechanisms supporting very long capacity of mycobacteria for resuscitation and further multiplication after prolonged survival in a dormant phase remain unclear. Using methods of 2D electrophoresis and MALDI-TOF analysis, in this study we characterized changes in the proteomic profile of *Mtb* stored for more than a year as dormant, non-replicating cells with a negligible metabolic activity, full resistance to antibiotics, and altered morphology (ovoid forms). Despite some protein degradation, the proteome of 1-year-old dormant mycobacteria retained numerous intact proteins. Their protein profile differed profoundly from that of metabolically active cells, but was similar to the proteome of the 4-month-old dormant bacteria. Such protein stability is likely to be due to the presence of a significant number of enzymes involved in the protection from oxidative stress (katG/Rv1908, sodA/Rv3846, sodC/Rv0432, bpoC/Rv0554), as well as chaperones (dnaJ1/Rv0352, htpG/Rv2299, groEL2/Rv0440, dnaK/Rv0350, groES/Rv3418, groEL1/Rv3417, HtpG/Rv2299c, hspX/Rv2031), and DNA-stabilizing proteins. In addition, dormant cells proteome contains enzymes involved in specific metabolic pathways (glycolytic reactions, shortened TCA cycle, degradative processes) potentially providing a low-level metabolism, or these proteins could be “frozen” for usage in the reactivation process before biosynthetic processes start. The observed stability of proteins in a dormant state could be a basis for the long-term preservation of *Mtb* cell vitality and hence for latent tuberculosis.

## Introduction

*Mycobacterium tuberculosis* (*Mtb*) is a most successful pathogen that may persist in a dormant state in a human body for decades and can reactivate to the active stage of disease after a long period of time (Flynn and Chan, 2001). Dormancy has been defined as a reversible state of low metabolic activity in which cells could survive for a long time without replication (Young et al., 2005). However, little is known about the biochemical processes which might occur in cells in the dormant state that provide long-lasting survival. Proteomic studies could potentially bring valuable information in this respect. Because only very little amounts of dormant *Mtb* cells can be recovered from the organs of infected individuals for such analysis, models which imitate the dormant state have been explored. Indeed, proteomic studies of dormancy models were performed using 2D electrophoresis (Florczyk et al., 2001; Betts et al., 2002; Rosenkrands et al., 2002; Starck et al., 2004; Devasundaram et al., 2016) and more advanced methods such us LC-MS/MS and SWATH (Albrethsen et al., 2013; Schubert et al., 2015). However, all known proteomic studies of dormant *Mtb* cells were performed on “short-term” models, such as the hypoxic Wayne model (formation of non-replicative form due to gradual depletion of oxygen in the growth medium) (Wayne, 1994) and the Loebel model based on starvation of cells in PBS buffer (Loebel et al., 1933), where the time of stress does not exceed 6 weeks. Moreover, the dormant cells obtained in these dormancy models don't mimic the true latent state *in vivo*, where cells are characterized by “non-culturability” (transient inability to grow on the non-selective solid media) and resistance to antibiotics (Khomenko and Golyshevskaya, 1984; Dhillon et al., 2004; Chao and Rubin, 2010). We have developed a model of the transition of *Mtb* cells into the dormant state based on the gradual acidification of the culture medium (Shleeva et al., 2011). The cells obtained in this model are characterized by a thickened cell wall, ovoid morphology, negligible metabolic activity, and resistance to antibiotics (Shleeva et al., 2011). In our study we explore proteomic profiling of *Mtb* dormant cells after 4 and 13 months of storage using 2D electrophoresis followed by MALDI-TOF analysis to characterize proteins (if any) of dormant cells that stored well after such a long period of time. The 2D electrophoresis method for proteome characterization was used in this study since this method allows the determining of intact proteins in the presence of the products of protein degradation which is highly possible after long storage.

## Materials and Methods

### Bacterial Strains, Growth Media, and Culture Conditions

Inoculum was initially growth from frozen stock stored at −70°C in 40% glycerine. *Mtb* strain H37Rv was grown for 8 days (up to OD_600_ = 2.0) in Middlebrook 7H9 liquid medium (Himedia, India) supplemented with 0.05% Tween 80 and 10% growth supplement ADC (albumin, dextrose, catalase) (Himedia, India). One milliliter of the initial culture was added to 200 ml of modified Sauton medium contained (per liter): KH_2_PO_4_, 0.5 g; MgSO_4_.7H_2_O, 1.4 g; L-asparagine, 4 g; glycerol, 2 ml; ferric ammonium citrate, 0.05 g; citric acid, 2 g; 1% ZnSO_4_.7H_2_O, 0.1 ml; pH 6.0–6.2 (adjusted with 1 M NaOH) and supplemented with 0.5% BSA (Cohn Analog, Sigma), 0.025% tyloxapol and 5% glucose. Cultures were incubated in 500 ml flasks contained 200 ml modified Sauton medium at 37°C with shaking at 200 rpm (Innova, New Branswick) for 30–50 days, and pH values were periodically measured. In log phase pH of the culture reached 7.5–8 and then decrease in stationary phase. When the medium in post-stationary phase *Mtb* cultures reached pH 6.0–6.2 (after 30–45 d of incubation for different experiments), cultures (50 ml) were transferred to 50 ml plastic tightened capped tubes and kept under static conditions, without agitation, at room temperature for up to 13 months post inoculation. At the time of transfer 2-(N-morpholino) ethanesulfonic acid (MES) was added in a final concentration 100 mM to dormant cell cultures to prevent fast acidification of the spent medium during long-term storage.

### Cyclic AMP Determination

Cell cultures at different stages of growth and storage were centrifuged at 13,000 g for 5 min. The cell pellets were treated with 1 ml 0.1 N HCl, heated at 95°C for 10 min and frozen immediately. The collected samples were disrupted by using a bead homogeniser FastPrep- 24, bacterial debris were removed by centrifugation, and aliquots of the supernatant were taken for the estimation of cAMP. To neutralize acidic sample Na_2_CO_3_ in concentration 2 M was used immediately before the samples were applied to the plates (Dass et al., 2008). cAMP levels were measured by ELISA using 96-well plates (96 Well ELISA Microplate, Greiner bio-one, Austria). Protein G (Imtek, Russia) (40 μg/ml) in PBS pH 7.4 was added to each well of 96-well plates and incubated for 2 h at the room temperature. Plates were washed using PBS containing 0.05% Triton X-100 (PBST). One hundred microliters of rabbit cAMP antibody (1:5,000) (GenScript, United States) and cAMP-HRP (1:20,000) (cAMP- peroxidase conjugate, GenScript, United States) was added to each well-followed by the addition of 50 μl of neutralized sample. After incubation for 2 h at room temperature microplates were washed with PBS. One hundred microliters of freshly prepared substrate contained 0.4 mM 3.3′,5.5′-tetramethylbenzidine (TMB, Sigma) in sodium citrate buffer (pH 4.0; 100 mM) with 3 mM H_2_O_2_ was added to each well and plates were incubated at room temperature for 30–40 min. The reaction was stopped by adding 100 μl 1M H_2_SO_4_. The results were registered at 450 nm with a Zenyth 3,100 microplate reader (Anthos Labtec Instruments, Austria). Three independent replicates were performed for each sample.

### Respiration, DCPIP Reduction

Endogenous respiratory chain activity (complex I) was determined by reduction of DCPIP (2,6-dichlorophenolindophenol) in the presence of menadione monitored spectrophotometrically at 600 nm. The reaction mixture (4 ml) contained 0.2 μmol 2,6-DCPIP, 0.6 μmol menadione, and 400 μl of the cell suspension in Sauton medium (pH 7.4).

### Microscopy

Phase–contrast epifluorescence microscopy was carried out on a Nikon eclipse Ni-U microscope, magnification 1,500×. Photos were taken using Nikon DS Qi2 camera (Japan).

### Viability Evaluation by MPN

Most probable number (MPN) assays of *Mtb* were performed in 48-well plastic plates (Corning) containing 1 ml special media for the most effective reactivation of dormant *Mtb* cells. This media contains 3.25 g nutrient broth dissolved in 1 liter of mixture of Sauton medium (0.5 g KH_2_PO_4_; 1.4 g MgSO_4_.7H_2_O; 4 g L-asparagine; 0.05 g ferric ammonium citrate; 2 g sodium citrate; 0.01% (w/v) ZnSO_4_.7H_2_O per liter pH 7.0), Middlebrook 7H9 liquid medium (Himedia, India) and RPMI (Thermo Fisher Scientific, USA) (1:1:1) supplemented with 0.5% v/v glycerol, 0.05% v/v Tween 80, 10% ADC (Himedia, India).

Mtb cells were serially 10-fold diluted in the reactivation medium. Appropriate five serial dilutions of *Mtb* cells (100 μl) were added to each well-contained the same medium in triplicate. Plates were incubated at 37°C with agitation at 130 rpm for 21 days. Wells with visible bacterial growth were counted as positive, and MPN values were calculated using standard statistical methods (de Man, 1974).

### Viability Evaluation by CFU

Bacterial suspensions were serially diluted in fresh Sauton medium with 0.05%, and three replicates of 10 μl samples from each dilution were spotted on Middlebrook 7H9 liquid medium (Himedia, India) supplemented 1.5% (w/v) agar and 10% (v/v) ADC (Himedia, India). Plates were incubated at 37°C for 30 days, and CFUs were counted. The lower limit of detection was 10 CFU/ml.

### Metabolic Activity Estimation

Cell metabolic activity was determined by incorporation of 1 μl of [5,6-^3^H]-uracil (1 μCi, 0.02 μmol) as well as L-[U-^14^C]-asparagine (4 MBq) into cells (1 ml). Cell suspension was incubated for 24 h at 37°C with agitation (for active cells) or at room temperature without agitation (for dormant cells). Cells (200 μl) were then harvested on glass microfiber GF/CTM filters (Whatman, UK) and washed with 3 ml 7% trichloroacetic acid followed by 3 ml absolute ethanol. Air-dried filters were placed in scintillation liquid (Ultima GoldTM, Perkin Elmer, USA), and the radioactivity incorporation was measured with a scintillation counter LS6500 (Beckman, USA).

### Sample Preparation for 2D Electrophoresis

Active and dormant cells obtained in four biological replicates were pooled (total volume 200 ml for each replicate, cell amount was ca 3.5 g wet weight) for 2D electrophoretic analysis. It is known that pooling allows to obtain an average expression of a particular protein which matches the mean expression of that protein after averaging of several individual replicates (Diz et al., 2009). The pooling should reduce influence of the technical factors (especially during the destruction of dormant cells and extraction of proteins) on the result of 2D electrophoresis. This approach has been previously used in mycobacterial proteomic studies (Betts et al., 2002; Trutneva et al., 2018). Bacteria were harvested by centrifugation at 8,000 g for 15 min and washed 10 times with a buffer containing (per liter) 8 g NaCl, 0.2 g KCl, and 0.24 g Na_2_HPO_4_ (pH 7.4). The bacterial pellet was re-suspended in ice-cold 100 mM HEPES (4-(2-hydroxyethyl)-1-piperazineethanesulphonic acid) buffer (pH 8.0) containing complete protease inhibitor cocktail (Sigma, USA) and PMSF (Phenylmethanesulphonyl fluoride) then disrupted with zirconium beads on a bead beater homogeniser (MP Biomedicals FastPrep-24) for 1 min, 5 times for active cells and 10 times for dormant cells. The bacterial lysate was centrifuged at 25,000 g for 15 min at 4°C. The supernatant was separated into membrane and cytosolic fractions using ultracentrifugation at 100,000 g for 2 h (Parish and Roberts, 2015). The membrane fraction was washed with HEPES buffer three times using ultracentrifugation. To isolate the proteins from membrane fraction extraction was performed using the strong anionic detergent sodium dodecyl sulfate (SDS) (2% w/v). The cytosolic fraction and membrane extract were precipitated using the ReadyPrep 2-D cleanup kit (BioRad, USA) to remove ionic contaminants such as detergents, lipids, and phenolic compounds from protein samples. This kind of precipitation allows resuspension of the protein pellet in isoelectric focusing buffer contains 8 M urea, 2 M thiourea, 10 mM 1,4-dithiothreitol (DTT), 2 mM TCEP (Tris(2-carboxyethyl)-phosphine-hydrochloride), 1% (w/v) CHAPS, 1% (w/v) Triton X-100, 1% (w/v) amidosulphobetaine-14 (ASB), and 0.4% (v/v) ampholytes (pH 3–10).

### Protein Amount Determination

Flores quantitative estimation of protein was used to check protein amount (Flores, 1978). Into 180 μl of the reaction mixture containing the bromophenol blue (0.0075%) dissolved in a solution of 15% ethanol and 2.5% glacial acetic acid was added 20 μl of sample in 100 mM Hepes (for cytosol) or in 100 mM Hepes in buffer (pH 8.0) contained 2% SDS dissolved (for membrane extracts). The absorbance at 610 nm was determined and adjusted to the calibration curve of the bovine serum albumin. Corresponding buffer was used for control.

### Two-Dimensional Electrophoresis

Isoelectric focusing was performed in a 5% acrylamide gel (30% (w/v) acrylamide/bisacrylamide, 8 M urea, 2% (v/v) ampholyte pH 3–10 and 4–6 (1:4), 1% (w/v) CHAPS, 1% (w/v) Triton X-100, 0.4% (w/v) ASB) using 2.4 mm ID glass tubes in a Tube Cell (Model 175, BioRad, USA) until 3,700 Vhrs were attained. One hundred micrograms of total protein amount of each sample was used for analysis. After focusing, gels were extracted from the glass tubes and fixed in equilibration buffer 1 (0.375 M Tris-HCl, pH 6.8, 2 M urea, 20% (v/v) glycerol, 2% (w/v) SDS, 2% (w/v) DTT) and equilibration buffer 2 (0.375 M Tris-HCl, pH 6.8, 2 M urea, 20% (v/v) glycerol, 2% (w/v) SDS and 0.01% (w/v) bromophenol blue) for 15 min each. Second-dimension separation was performed as described by O'Farrell (O'Farrell, 1975) in large format (20 × 20 cm), 1.5 mm thick 12% SDS-PAGE gels in standard Tris-glycine buffer in a PROTEAN II xi cell for vertical electrophoresis (BioRad, USA). The gels were stained by Coomassie CBBG-250 (Roti-Blue Carl Roth, Germany) followed by silver staining (https://www.alphalyse.com/wp-content/uploads/2015/09/Silver-staining-protocol.pdf).

The gels images were captured using Syngene G:BOX Gel & Blot Imaging Systems (Syngene, UK). Gel images stained by Coomassie were analyzed using TotalLab TL120 software to calculate spot density.

Each visible protein spot was excised manually from the gel and analyzed using MALDI-TOF. The MS/MS data obtained from MALDI-TOF were subjected to a Mascot Protein Database (MSDB) search to identify proteins. Proteins with coverage < 10% were not further considered. Protein functional roles for *Mtb* were obtained from the Mycobrowser database (https://mycobrowser.epfl.ch). Each sample for 2D analysis was performed in two technical replicates.

### Protein Identification by MALDI-TOF

All fractions excised from 2D electrophoresis slab gels were hydrolyzed by trypsin digestion. The extracted tryptic peptides were analyzed by MALDI-TOF as described previously, with some modifications. A sample (0.5 μl) was mixed with the same volume of 20% (v/v) acetonitrile solution containing 0.1% (v/v) trifluoroacetic acid and 20 mg/ml 2,5-dihydroxybenzoic acid and then air-dried. Mass spectras were obtained on a Reflex III MALDI-TOF mass spectrometer with a UV laser (336 nm) in positive-ion mode in the range of 500–8,000 Da. Calibration was performed in accordance with the known peaks of trypsin autolysis.

For MS/MS analysis, the mass spectra of fragments were recorded with a Bruker Ultraflex MALDI-TOF mass spectrometer in tandem mode (TOF-TOF) with detection of positive ions. The proteins were identified using Mascot software in Peptide Fingerprint mode (Matrix Science, Boston, MA, USA). The accuracy of the mass measurement MH+ was 0.01% (with a possibility of modifying cysteine by acrylamide and methionine oxidation). Raw data and search results could be found on PeptideAtlas: http://www.peptideatlas.org/PASS/PASS01450.

## Results

Active cells for proteome analysis were obtained in the early stationary phase after 10 days of cell growth under agitation (culture “A”) in standard Sauton medium (Connell, 1994). Dormant *Mtb* cells in the prolonged stationary phase were obtained by gradual acidification of the medium according to a published protocol (Shleeva et al., 2011) with some minor modifications (see M&M). Dormant cells were kept in plastic-capped tubes to avoid evaporation in the dark at room temperature for 4.5 months (culture “D1”) and 13 months (culture “D2”). Under these conditions cells remain aerobic as the methylene blue did not decolorize and hence oxygen was not completely depleted (Shleeva et al., 2011). In addition, cytochrome composition of respiratory chain for culture D1 did not differ from active cells (Nikitushkin, personal communication) which indicates aerobic condition in contrast to Wayne cells where increased *bd* cytochrome oxidase as a terminal acceptor under oxygen depletion has been found (Kana et al., 2001; Shi et al., 2005). The estimated viability of stored dormant cells by CFU was about 10^4^ cells/ml for D1 and zero for D2 mycobacteria (Table 1). The MPN (Most probable number) assay (estimation of viability in liquid medium) revealed a viable cell number higher than CFU, reflecting the reversible “non-culturablity” of these dormant cells on the solid media after this storage period (Table 1). According to the biochemical studies, dormant cells did not show transcriptional (by ^3^H-uracil incorporation) and translational activity (by ^14^C-asparigine incorporation), and they were characterized by a significant decrease in the activity of endogenous DCPIP (2,6-dichlorophenolindophenol) reduction reflecting respiratory activity (complex I) (Table 1).

**Table 1:** Some properties of active and dormant *Mtb* cells.

**Characteristic**	**Active cells**	**Dormant cells 4.5 months (D1)**	**Dormant cells** **13 months (D2)**
CFU, cells/ml	(5 ± 2) × 10^7^	(1 ± 0.5) × 10^4^	0
MPN, cells/ml	1.2 × 10^8^-mean 3.7 × 10^7^-low^*^ 4.2 × 10^8^-high^*^	9.3 × 10^7^-mean 1.8 × 10^7^-low^*^ 4.2 × 10^8^-high^*^	1.5 × 10^6^-mean 4.5 × 10^5^-low^*^ 4.2 × 10^6^-high^*^
H^3^- Uracil inc. rate, CPM/mg wet cell weight	3,623 ± 52	0	0
cAMP, pmol/mg wet cell weight	124 ± 4	2 ± 1	0
^14^C-asparigine inc. rate CPM/mg wet cell weight	340 ± 20	0	0
DCPIP reduction, nmol DCPIP min^−1^ mg^−1^ wet cell weight	0.11 ± 0.03	0.016 ± 0.003	0
Protein amounts in cytosol, mg/g wet cell weight	3.6 ± 0.2	2.13 ± 0.2^**^	2.55 ± 0.2^**^
Protein amounts in membrane fraction, μg/g wet cell weight	76.2 ± 5	39 ± 5^**^	20.7 ± 5^**^

* *95 percent confidence intervals*.

** *Dormant cells were 10 times washed before protein determination*.

We also checked the intracellular concentration of cAMP. Previously we found that the transition of *Mtb* to the dormant “non-culturable” state correlates with a decrease in the cAMP concentration (Shleeva et al., 2017a). Alternatively, cAMP concentration increases in *Msm* cells under resuscitation (Shleeva et al., 2013). Accordingly, we found a decrease in the cAMP concentration in *Mtb* cells after 4.5 months of storage which accompanied the significant developing of “non culturability” (Table 1).

**Figure 1:**
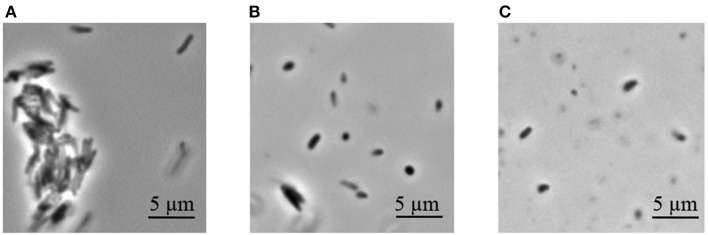
Phase contrast microscopy of *M. tuberculosis* cells (magnification 1.500). **(A)** Active cells in early stationary phase. **(B)** Dormant cells after 4.5 months storage at room temperature. **(C)** Dormant cells after 13 months storage.

Dormant cells were washed 10 times using PBS to remove dead cells. This approach results in ~60% intact cells in the population according to PI (propidium iodide) staining (not shown). Such cells appeared small and ovoid in comparison to the rod-shaped cells typical of multiplying bacteria (Figure 1). These dormant cells contained less protein per mg wet cell weight in comparison to active cells. This was more evident for the membrane fraction than for the cytoplasm (Table 1). Obtained dormant cells were used for proteomic analysis. In each experiment the protein amount used for the first dimension was identical for both types of cells, although the total amount of protein isolated from active cells was different.

**Figure 2:**
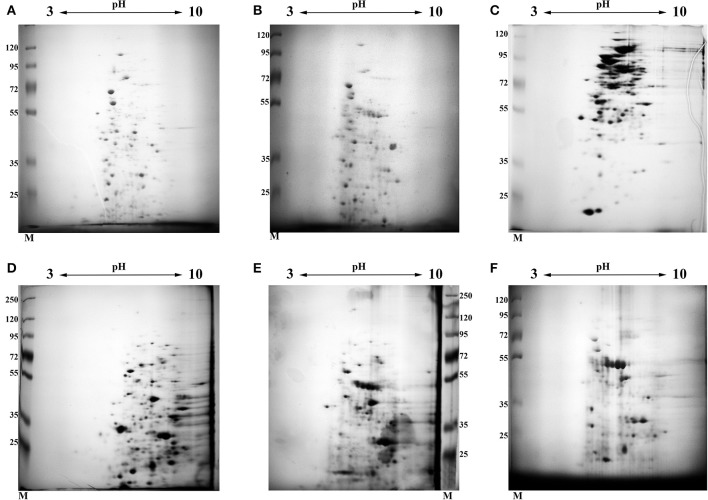
2D electrophoresis of different fractions obtained from active and dormant *M. tuberculosis* cells. Each gel was stained by Coomassie followed by silver staining. **(A)** Cytosol fraction of active, early stationary phase cells. **(B)** Cytosol fraction of dormant cells after 4.5 months storage at room temperature (D1). **(C)** Cytosol fraction of dormant cells after 13 months storage at room temperature (D2). **(D)** Membrane fraction extracted by SDS of active, early stationary phase cells. **(E)** Membrane fraction extracted by SDS of dormant cells after 4.5 months storage at room temperature (D1). **(F)** Membrane fraction extracted by SDS of dormant cells after 13 months storage at room temperature (D2). The gel photo represents one out of two identical technical replicates.

In order to elucidate and characterize the pool of proteins which are presented in “early” –(D1, 4.5 months of storage when significant decrease in metabolic activity judged by uracil incorporation was found) and “late” (13 month of storage D2) dormant cells, we analyzed the protein composition of different fractions (cytosol and membranes) by 2D electrophoresis. The results of these experiments are shown in Figure 2 (2D photo). Manual excision of each spot from the gel followed by MALDI TOF allowed us to uncover a total of 21,703 peptides (12,318 cytosol + 9,385 membranes) in all 3 types of cells including protein repeats in the different spots. According to the Mascot database those peptides belonged to 1,131 individual proteins (629 for cytosol and 502 for membranes) including repeating proteins in different types of cells (Table S1). Out of these, 446 could be linked to proteins with a different Rv (Uniprot) number (305 for cytosol and 243 for membranes). Each spot contained from 1–4 different proteins (on average 2.2 proteins for all types of cells) in one spot.

**Figure 3:**
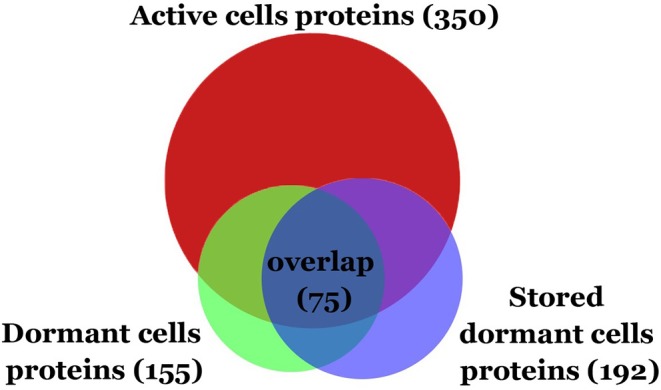
Venn diagram showing the protein overlap between active, dormant (D1), and stored dormant cells (D2).

Comparative analysis revealed the reduction of protein diversity (350 proteins for active cells, 155 for D1 and 192 for D2 cells) (Figure 3), which could be apparently associated with protein degradation during cell transition to the dormant state followed by a long storage. Most significant degradation of proteins was found for cytosol fraction (243 proteins for active cells, 77 for D1 and 92 for D2 cells). In contrast, membrane proteins exhibited more stability (108 proteins for active cells, 79 for D1 and 102 for D2 cells). Protein degradation was visible in the increasing of a front line upon electrophoresis containing evidently degraded material (Figure 2). Most likely degradation of proteins into peptides that give a contribution to total protein measuring is responsible for the comparably small difference in protein amount per mg of cells vs. active cells found especially in the cytosolic fraction (Table 1).

Only small changes in proteins diversity could be found between early dormant cells and stored dormant cells and more than 50% of the protein is shared between two types of cells (Figure 3). This comparison reveals a pool of highly stable proteins in dormant state. At the same time, old dormant cells proteome (D2) contained proteins that are detected neither in the active nor in the early dormant proteome (Table S2). Apparently, these are stable proteins but in minor abundance in active cells. 53 proteins in D1, which are not presented in the dormant D2 proteome, were evidently degraded during the late phase of storage. From these, 43 proteins were found neither in active nor in stored dormant culture proteomes, probably reflecting their importance in the transition from multiplying to dormant state.

Identified proteins were ranked by their representation in the whole proteome, on the basis of the spot density (Table S1). This analysis allows comparing individual protein abundance in different cell types under conditions when total amount of protein per cell is not equal (Table 1).

A similar approach has been used previously for the comparative proteomic analysis of active and dormant *Msm* cells (Trutneva et al., 2018).

This analysis allows to find a cohort of proteins with substantially changed abundance in dormant vs. active cells [totally 139 increased proteins and 248 decreased proteins (Table S3)]. Remarkably, that majority of proteins found in 10 most abundant spots in D2 culture are more abundant or even ≪unique≫ in comparison with active culture (Table 2). We also analyzed the proteins with the aim of determining their stability after long storageand to elucidate metabolic pathways in which they could participate.

**Table 2:** Proteins found in 10 most abundant spots in D2 proteome profile.

**Fraction**	**Product**	**Gene**	**Place, active**	**Place, D2**
Membrane	Probable iron-regulated elongation factor TU Tuf (EF-TU)	Rv0685	2	1
Cytosol	Probable 5-methyltetrahydropteroyltriglutamate–homocysteine methyltransferase MetE (methionine synthase, vitamin-B12 independent isozyme)	Rv1133c	85	1
Cytosol	Transketolase Tkt (TK)	Rv1449c	N/D	1
Cytosol	Malate synthase G GlcB	Rv1837c	134	1
Cytosol	Catalase-peroxidase-peroxynitritase T KatG	Rv1908c	85	1
Cytosol	Probable chaperone protein DnaK (heat shock protein 70) (heat shock 70 kDa protein) (HSP70)	Rv0350	5	2
Cytosol	60 kDa chaperonin 2 GroEL2 (protein CPN60-2) (GroEL protein 2) (65 kDa antigen) (heat shock protein 65) (cell wall protein A) (antigen A)	Rv0440	3	2
Membrane	Conserved 35 kDa alanine rich protein	Rv2744c	9	2
Cytosol	Maltokinase Mak	Rv0127	N/D	3
Membrane	Isoniazid inductible gene protein IniB	Rv0341	N/D	3
Membrane	Conserved protein	Rv1232c	41	3
Cytosol	Glutamine synthetase GlnA1 (glutamine synthase) (GS-I)	Rv2220	122	3
Cytosol	Probable adenosylhomocysteinase SahH (S-adenosyl-L-homocysteine hydrolase) (adohcyase)	Rv3248c	N/D	3
Cytosol	10 kDa chaperonin GroES (protein CPN10) (protein GroES) (BCG-a heat shock protein) (10 kDa antigen)	Rv3418c	N/D	4
Membrane	Probable bifunctional protein acetyl-/propionyl-coenzyme A carboxylase (alpha chain) AccA3: biotin carboxylase + biotin carboxyl carrier protein (BCCP)	Rv3285	83	5
Membrane	60 kDa chaperonin 1 GroEL1 (protein CPN60-1) (GroEL protein 1)	Rv3417c	48	6
Cytosol	Probable iron-regulated phosphoenolpyruvate carboxykinase [GTP] PckA (phosphoenolpyruvate carboxylase) (PEPCK)(pep carboxykinase)	Rv0211	130	7
Membrane	Probable short-chain type oxidoreductase	Rv0484c	N/D	7
Membrane	Possible ketoacyl reductase	Rv1544	N/D	7
Membrane	DNA-binding protein HU homolog HupB (histone-like protein) (HLP) (21-kDa laminin-2-binding protein)	Rv2986c	N/D	7
Cytosol	Probable acetohydroxyacid synthase IlvX (acetolactate synthase)	Rv3509c	83	8
Cytosol	Probable NAD(P) transhydrogenase (subunit alpha) PntAa [first part; catalytic part] (pyridine nucleotide transhydrogenase subunit alpha) (nicotinamide nucleotide transhydrogenase subunit alpha)	Rv0155	94	9
Membrane	Probable succinate dehydrogenase [iron-sulfur subunit] (succinic dehydrogenase)	Rv0247c	12	9
Cytosol	Probable succinyl-CoA synthetase (beta chain) SucC (SCS-beta)	Rv0951	119	9
Cytosol	Probable phosphoglycerate kinase Pgk	Rv1437	94	9
Membrane	Probable catechol-O-methyltransferase	Rv1703c	N/D	9
Membrane	Conserved protein	Rv3205c	N/D	9
Membrane	Probable O-antigen/lipopolysaccharide transport ATP-binding protein ABC transporter RfbE	Rv3781	N/D	9
Membrane	Probable short-chain type dehydrogenase/reductase	Rv0148	N/D	10
Membrane	Periplasmic superoxide dismutase [Cu-Zn] SodC	Rv0432	5	10
Cytosol	Probable citrate synthase I GltA2	Rv0896	N/D	10

*In the columns marked as “place,” proteins were arranged according to their spot density. See also M&M. Proteins with increased abundance in D2 cells vs. active cells (place for active cells proteome minus place for D2 > 10) are highlighted including proteins which were virtually absent in the active cells proteome (marked as “ND”)*.

Such analysis of D2 proteins and their sorting according to their participation in particular metabolic reactions was represented in Table S4. Among them quite a few proteins were found to belong to the tricarboxylic acid (TCA) cycle (in contrast to the active cell proteome where all components were found). Namely, enzymes which facilitate the conversion of oxaloacetate to succinate via fumarate (mdh/Rv1240, fum/Rv1098, succinate dehydrogenase/Rv0247, Rv0248), compose the reductive branch of the TCA cycle which could potentially be used for the TCA cycle functioning in the opposite direction with formation of succinate as an end product (Zimmermann et al., 2015). The D2 proteome contains enzymes involved in the glycolytic pathway (glucose-6-phosphate isomerase/Rv0946, fructose-bisphosphate aldolase/Rv0363, phosphoglycerate kinase/Rv1437, enolase/Rv1023, pyruvate kinase/Rv1617), biosynthesis of fatty acids, cell wall and amino acid interconversion. A significant proportion (18 proteins) of the D2 proteome were found to participate in the hydrolysis of lipids, proteins and amino acids (Table S4).

Among the 192 proteins found in D2 cells a substantial number (19 proteins) belong to enzymes involved in defense mechanisms. Enzymes with catalase/peroxidase/superoxide dismutase activities (katG/Rv1908, sodA/Rv3846, sodC/Rv0432, bpoC/Rv0554) were presented in D2 cells. DNA binding histone-like protein (hupB/Rv2986) (Table 2) is capable of stabilizing DNA and prevents its denaturation under stress conditions (Enany et al., 2017). Previously it was found that HupB ortholog in *Msm* (Hlp) can provide compactization of the nucleoid during dormancy (Anuchin et al., 2010). It is interesting that a major protein found in the membrane fraction in D2 cells is Rv0341(iniB, Table 2) with unknown function that could also modify DNA topology due to its ability to interact with DNA (Shleeva et al., 2018) via a DNA-binding domain presented in the molecular structure according to Uniprot data base annotation.

The thioredoxin system of Rv3913/Rv3914, a well-known antioxidant defense system that participates in the virulence-determining mechanism in *Mtb* (Budde et al., 2004) was found “uniquely” in the proteome of dormant cells. In addition, the thioredoxin system and the protein with unknown function Rv2466c (“unique” for dormant cells as well) is controlled by SigH sigma factor, which is involved in the adaptation of *Mtb* to heat shock, oxidative and nitrosive stress (Raman et al., 2001). However, neither SigH nor other sigma factors were found, possibly due to their low concentrations in the cell.

Several chaperone proteins were found to be highly presented in D2 (dnaJ1/Rv0352, htpG/Rv2299, groEL2/Rv0440, dnaK/Rv0350, groES/Rv3418, groEL1/Rv3417, HtpG/Rv2299c, hspX/Rv2031). It is not a surprise that Heat shock protein (HspX/Rv2031c), a member of the DosR regulon, was found to be increased in the dormant cells, since its significant accumulation was found in all other dormancy models (Florczyk et al., 2001; Betts et al., 2002; Rosenkrands et al., 2002; Starck et al., 2004; Mishra and Sarkar, 2015; Devasundaram et al., 2016). Apart from HspX, among 48 proteins belonging to the DosR regulon, several universal stress proteins (Rv2623, Rv1996, Rv2624) and several proteins with unknown functions (Rv2004, Rv2629) were only found.

Secreted antigen 85-B FbpB/Rv1886c that possesses mycolyltransferase activity required for the biogenesis of trehalose dimycolate (cord factor), a dominant structure necessary for maintaining cell wall integrity (Nguyen et al., 2005) was found in D2.

A number of proteins that participate in transcription and translation processes were represented in D1/D2 despite negligible activity of these processes under dormancy (Table S1). Other proteins involved in different metabolic reactions, transport activity and transcriptional regulation were shown in Table S1. It is interesting that despite the almost zero level of respiratory chain activity [by DCPIP (Table 1) and by methylene blue (Shleeva et al., 2011) reduction] its components as well as H^+^-ATPase (Rv1308, Rv1309, Rv1310) were found in dormant cells.

Among proteins with increased abundance in dormant cells (Table S3) mycobacterial persistence regulator MprA/Rv0981, the response regulator and part of two-component histidine-kinase system was found in D1. Under stress conditions, MprAB induces sigE transcription, which leads to an increase in the level of relA and, as a consequence, an increase in the level of (p)ppGpp eliciting the stringent response. Such a mechanism, in which the cascade of reactions begins with MprAB activation, is a specific pathway for mycobacteria (e.g., *M. smegmatis*; Sureka et al., 2008), since in other bacteria the first stage is strictly associated with the synthesis of relA (Magnusson et al., 2005). Expression of Rv0981 on the transcriptional level has been found in non-culturable deep dormant *Mtb* cells (Ignatov et al., 2015) whilst short-term dormant Wayn's cells did not show changes in the expression of this gene neither on transcriptional (Galagan et al., 2013) nor proteomic level (Schubert et al., 2015).

Another two-component PhoPR system regulates the *espA* gene cluster which is essential for virulence (Pang et al., 2013; Zhang et al., 2018). In our case, the level of phoP/Rv0757 increased in D1 and D2. It is known that this system is activated when cells enter a low pH environment resulting in the activation of a cluster of genes that help the cell to cope with oxidative stress (García et al., 2018). PhoPR has recently been shown to be a negative regulator of the DosRS (DevRS) system (Vashist et al., 2018). Perhaps that is a reason for the absence of the DevR regulator itself and other proteins belonging to DosR regulon in our model. This is in line with unchanged regulation of the expression of Rv0757 on both transcriptional (Galagan et al., 2013) and proteomic level (Schubert et al., 2015) in short-term Wayne dormancy. Whilst in more prolonged hypoxic conditions (enduring response) transcription of this gene was found to be up-regulated resulting in suppression of DosR regulon expression (Rustad et al., 2008).

A transcription regulator found upregulated in dormant cells is Diviva family protein Wag31 (Rv2145), which regulates cell shape and cell wall synthesis in *Mtb* through a molecular mechanism by which the activity of Wag31 can be modulated in response to environmental signals (Kang et al., 2008). In addition, Wag31 is one substrate of PknA and PknB kinases (Lee et al., 2014). Increased expression of Rv2145 was found on transcriptional level in non-culturable deep dormant *Mtb* cells (Ignatov et al., 2015) and in prolonged starvation model on proteomic level (Albrethsen et al., 2013). However it did not show expression changes in anaerobic cells (Rustad et al., 2008; Galagan et al., 2013; Schubert et al., 2015).

Elongation factor TU (Ef-Tu)/Rv0685, which is normally responsible for the selection and binding of the cognate aminoacyl-tRNA was found in large amounts in all types of cells. The activity of Ef-Tu is dependent on its interaction with GTP which, in turn, depends on Ef-Tu phosphorylation by protein kinases. Such phosphorylation results in a reduction in protein synthesis followed by a reduction of cell growth (Sajid et al., 2011). High amounts of Ef-Tu while phosphorylated could probably cause protein synthesis arrest during the dormant phase. Dephosphorylation of Ef-Tu under resuscitation of dormant *Mtb* cells would result in protein synthesis beginning.

Comparative proteomic analysis revealed that the diversity of active transporters dramatically decreased in dormant cells (Table S3). The proteomic profile of the late stage of dormancy (D2) contains only a few transporters comparative to active cells that contain protein, amino acids, oligopeptide, sugar and ion transporters. Similarly, the level of cAMP synthase (Rv1264) was found significantly decreased in D1 and D2 proteomes (Table S3). It was established that level of cAMP negatively correlates with storage time of dormant cells (Table 1).

The enzymes involved in biosynthesis of cell wall-found in dormant cells proteome were characterized by decreased abundance (Table S3).

## Discussion

Despite long-term storage the dormant *Mtb* cells' proteome remained enriched with a large diversity of proteins. This is the first investigation where the proteome of dormant *Mtb* cells stored for over a year was examined. Other dormant cells' proteome studies were carried out on cells after 20 days of hypoxic conditions (Schubert et al., 2015) or 6 weeks during starvation (Albrethsen et al., 2013). These short-term proteomic studies had a large overlap between active and dormant cell proteins. Thus, 69 and 66% of the first 200 most represented proteins were identical in those two models, respectively. However, in the present study we have a greater difference in the proteomes of active vs. dormant cells, and the overlap is only 47%. This discrepancy is evidently associated with a deeper dormancy state developed after more than 1 year of storage where cells, in contrast to “short” models, are characterized by “non-culturability” and negligible metabolic activity (Table 1). Presumably, long storage of cells results in the selection of the proteins which are most stable during the long period of dormancy and are not degraded within D2 cells and were thus available for analysis.

Comparative analysis of the 200 most abundant proteins in our dormancy model with proteins in Wayne (Schubert et al., 2015) and Loebel (Albrethsen et al., 2013) dormancy models reveals 58 “consensus” proteins (Table 3, Table S5). Eleven proteins from this list are involved in defense mechanisms and 14 proteins participate in central metabolic pathways (tricarboxylic acid cycle, glycolysis, respiratory chain, and ATPase). DNA-dependent RNA polymerase (alpha and beta chain) as well as elongation factors (Tu/Rv0685, Ts/Rv2889c) also presented in the 200 most abundant proteins shared between the three dormancy models. Obviously, these “consensus” proteins have unique stability in all dormant models despite different inducing factors and may play special roles in the maintaining of cell viability under stress conditions. It is interesting that proteins belonging to the DosR regulon were poorly represented both in D1 and D2, which highlights the difference between the Wayne anaerobic model and the model used in this study. This is also true for the Wayne vs. the Loebel model (Albrethsen et al., 2013). Evidently, due to the low metabolic activity of dormant cells, a large number of proteins (enzymes) found in the late phase of dormancy (D2) are not functionally active. For example, ATPase is unable to perform the synthesis of ATP because of the extremely low activity of the respiratory chain which results in low ATP level (Shleeva et al., 2011). The same is applicable for enzymes involved in transcription and translation. At the same time, such inactive D2 proteins can be considered as a reserve which can be used in the early stages of reactivation before transcription and protein synthesis *de novo*. Indeed, the beginning of the transcription takes place not at the first moment of reactivation of long-stored dormant cells, but much later, 4 days after reactivation (not shown). On the other hand, some D2 proteins could be functionally active, providing some level of metabolism maintenance. In addition, some degradative enzymes could be potentially active to provide metabolic substrates to maintain some level of metabolism and cell vitality (“catabolic survival”). For example, we found that in *Msm* dormant cells, trehalose could be used as a source of glucose assimilated in the glycolytic pathway (Shleeva et al., 2017b), the enzymes of which were found in D2 *Mtb* proteome. We cannot exclude that the reductive branch of the TCA cycle is functional in dormant *Mtb* resulting in extracellular accumulation of succinate that makes it possible to oxidize reductive equivalents formed in glycolysis (Zimmermann et al., 2015).

**Table 3:** “Consensus” proteins shared between the 3 *Mtb* dormancy models found in the first 200 most abundant.

**Gene number**	**Product**
Rv1908c	Catalase-peroxidase-peroxynitritase T KatG
Rv1837c	Malate synthase G GlcB
Rv1133c	Probable 5-methyltetrahydropteroyltriglutamate–homocysteine methyltransferase MetE (methionine synthase, vitamin-B12 independent isozyme)
Rv0685	Probable iron-regulated elongation factor TU Tuf (EF-TU)
Rv1449c	Transketolase Tkt (TK)
Rv0440	60 kDa chaperonin 2 GroEL2
Rv2744c	Conserved 35 kDa alanine rich protein
Rv0350	Probable chaperone protein DnaK (heat shock protein 70) (heat shock 70 kDa protein) (HSP70)
Rv2220	Glutamine synthetase GlnA1 (glutamine synthase) (GS-I)
Rv3248c	Probable adenosylhomocysteinase SahH (S-adenosyl-L-homocysteine hydrolase) (adohcyase)
Rv3418c	10 kDa chaperonin GroES (protein CPN10) (protein GroES) (BCG-a heat shock protein) (10 kDa antigen)
Rv3285	Probable bifunctional protein acetyl-/propionyl-coenzyme A carboxylase (alpha chain) AccA3: biotin carboxylase + biotin carboxyl carrier protein (BCCP)
Rv3417c	60 kDa chaperonin 1 GroEL1 (protein CPN60-1) (GroEL protein 1)
Rv0211	Probable iron-regulated phosphoenolpyruvate carboxykinase [GTP] PckA
Rv0951	Probable succinyl-CoA synthetase (beta chain) SucC (SCS-beta)
Rv0896	Probable citrate synthase I GltA2
Rv1074c	Probable beta-ketoacyl CoA thiolase FadA3
Rv0363c	Probable fructose-bisphosphate aldolase Fba
Rv1617	Probable pyruvate kinase PykA
Rv1017c	Probable ribose-phosphate pyrophosphokinase PrsA (phosphoribosyl pyrophosphate synthetase)
Rv1310	Probable ATP synthase beta chain AtpD
Rv2280	Probable dehydrogenase
Rv2145c	Diviva family protein Wag31
Rv3028c	Probable electron transfer flavoprotein (alpha-subunit) FixB (alpha-ETF) (electron transfer flavoprotein large subunit) (ETFLS)
Rv1886c	Secreted antigen 85-B FbpB (85B) (antigen 85 complex B) (mycolyl transferase 85B) (fibronectin-binding protein B) (extracellular alpha-antigen)
Rv1094	Possible acyl-[acyl-carrier protein] desaturase DesA2 (acyl-[ACP] desaturase) (stearoyl-ACP desaturase)
Rv1023	Probable enolase Eno
Rv1475c	Probable iron-regulated aconitate hydratase Acn (citrate hydro-lyase) (aconitase)
Rv0815c	Probable thiosulfate sulfurtransferase CysA2
Rv3246c	Two component sensory transduction transcriptional regulatory protein MtrA
Rv1436	Probable glyceraldehyde 3-phosphate dehydrogenase Gap (GAPDH)
Rv3224	Possible iron-regulated short-chain dehydrogenase/reductase
Rv2996c	Probable D-3-phosphoglycerate dehydrogenase SerA1 (PGDH)
Rv0860	Probable fatty oxidation protein FadB
Rv3841	Bacterioferritin BfrB
Rv0831c	Conserved protein
Rv0242c	Probable 3-oxoacyl-[acyl-carrier protein] reductase FabG4
Rv2971	Probable oxidoreductase
Rv3280	Probable propionyl-CoA carboxylase beta chain 5 AccD5 (pccase) (propanoyl-CoA:carbon dioxide ligase)
Rv3457c	Probable DNA-directed RNA polymerase (alpha chain) RpoA
Rv1308	Probable ATP synthase alpha chain AtpA
Rv3846	Superoxide dismutase [FE] SodA
Rv3274c	Probable acyl-CoA dehydrogenase FadE25
Rv1630	30S ribosomal protein S1 RpsA
Rv2780	Secreted L-alanine dehydrogenase Ald (40 kDa antigen) (TB43)
Rv3914	Thioredoxin TrxC (TRX) (MPT46)
Rv0667	DNA-directed RNA polymerase (beta chain) RpoB (transcriptase beta chain)
Rv2140c	Conserved protein TB18.6
Rv0468	3-hydroxybutyryl-CoA dehydrogenase FadB2
Rv3596c	Probable ATP-dependent protease ATP-binding subunit ClpC1
Rv2334	Cysteine synthase a CysK1
Rv0462	Dihydrolipoamide dehydrogenase LpdC
Rv0684	Probable elongation factor G FusA1 (EF-G)
Rv0632c	Probable enoyl-CoA hydratase EchA3
Rv2031c	Heat shock protein HspX (alpha-crystallin homolog) (14 kDa antigen) (HSP16.3)
Rv2889c	Probable elongation factor Tsf (EF-ts)
Rv2215	DlaT, dihydrolipoamide acyltransferase, E2 component of pyruvate dehydrogenase
Rv2299c	Probable chaperone protein HtpG (heat shock protein)

One of the most remarkable features of long-stored dormant cells is their enrichment by enzymes that protect cells against oxidative stress (superoxide dismutases, catalases, and peroxidases) and prevent protein aggregation (chaperones). In addition, the found DNA binding proteins (hupB/Rv2986, iniB /Rv0341) could possibly provide stabilization of DNA therefore contributing to overall cell vitality and preventing its denaturation under stress conditions.

Previously, we performed similar experiments with dormant *Msm* cells obtained after gradual acidification of the media in a prolonged stationary phase (Trutneva et al., 2018).

Upon comparison of proteome profile of dormant Mtb and *Msm* cells, we may see both similarities and differences in protein composition. The most evident difference is significant reduction of total amount of proteins found in *Mtb* “dormant proteome” (44–55% from “active proteome”) in comparison with dormant *Msm* (96% from active proteome).

Comparison of protein composition of dormant cells for two species reveal significant amount of enzymes in *Msm* participated in different metabolic pathways which normally belong to active metabolism (biosynthesis of aminoacids, purines, and pyrimidines, fatty acids, trehalose, porphyrines, cell wall, transport, and replication processes). It is highly unlikely that those enzymes and corresponding processes could take place under non-replicative state with low metabolic activity. We suggested that such proteins are comparatively stable during transition and storage and could be used under resuscitation, that makes dormant *Msm* easy to recover and therefore to be culturable (Trutneva et al., 2018) in contrast to *Mtb*.

Whilst there is a significant difference in protein diversity in two species in dormancy (dormant D2 *Mtb* proteome profile contains only 27% orthologs found in *Msm* proteome profile) we may find a cohort of functionally identical proteins in the two proteomes. Considering annotated protein orthologists in two species (280 dormant *Msm* and 192 in D2 *Mtb* culture), this cohort contains 78 proteins belonging to central metabolic pathways like glycolysis and the TCA cycle. The overlapped *Msm/Mtb* dormant proteome profile is similarly enriched with chaperones and proteins that provide degradative reaction and defense mechanisms against stresses (Table S6). Such cohort of functionally identical proteins could maintain “minimal metabolism” in dormant state providing cell survival and stress defense without multiplication.

Thus, the two studies reveal a partially similar response of *Mtb* and its non-pathogenic relative *Msm* to adaptation to dormancy at the proteome level making the found proteomic changes general for the dormant state in mycobacteria.

In summary, this study demonstrates that *Mtb* cells under long-term storage in a dormant, “non-culturable” state contain significant amounts of stable proteins with different functional activities. Despite the mechanisms of such unique stability in the absence of protein synthesis being unclear, it is evident that the specific enzymes and proteins provide a “defending shell' for other proteins contributing to overall cell stability and vitality. The further study of the cohort of long-term protein survivors would provide a clue for the mechanisms of *Mtb* persistence in the host organism and finding of new targets for the development of new drugs to combat latent tuberculosis.

## Data Availability Statement

The datasets generated for this study can be found in: http://www.peptideatlas.org/PASS/PASS01450.

## Author Contributions

AK and KT conceived and designed the experiments, analyzed the data, and wrote the manuscript. MS, KT, GD, and GV performed the experiments. KT prepared figures and graphs. All authors read and approved the final manuscript.

### Conflict of Interest

The authors declare that the research was conducted in the absence of any commercial or financial relationships that could be construed as a potential conflict of interest. The reviewer AM declared a shared affiliation, with no collaboration, with the authors to the handling editor at time of review.

## Supplementary Material

Table S1 The proteins found in cytosol and membrane fractions of active and two types of dormant *M. tuberculosis* cells. Active cells were harvested from early stationary phase; dormant cells were obtained after gradual acidification in stationary phase followed by 4.5 months storage at room temperature (D1) and stored dormant cells were obtain after 13 months of storage (D2). 2D electrophoresis and protein analysis for cytosol and membrane (SDS extracts) were performed as described in the M&M. Density estimation of each protein spot was performed in two technical replicates of the pooled samples obtained from 4 independent biological replicates for active and dormant bacteria. The average results are shown. The relative error for density values of each spot did not exceed 5%. In the columns marked as “place,” proteins were arranged according to their spot density from highest (1) to lowest (159 for active, 92 for D1 cells and 75 for D2 cells) representation in the proteome. Proteins which were virtually absent in the other proteome marked as “ND.” If a protein with a particular accession number is found in several spots, the corresponding rank was assigned for a spot with maximum density. Column marked as “Mass values matched” shows a number of experimentally found peptides matched with theoretically predicted peptides for particular protein. Column marked as “coverage” shows percent coverage calculated by dividing the number of amino acids in all found peptides by the total number of amino acids in the entire protein sequence. Protein functional roles for Mtb were obtained from the Mycobrowser database (https://mycobrowser.epfl.ch).

Table S2 The proteins found only in stored *Mtb* dormant cells proteome (13 months), but not in other types of cells.

Table S3 Proteins with substantially changed abundance in dormant cells (D2) proteome. Proteins with increased and decreased abundance in D2 cells vs. active cells (place for active cells proteome minus place for D2 > |10|) including proteins which were virtually absent in the other cells proteome (marked as “ND”) are shown.

Table S4 Distribution of the proteins found in the proteomic profile of stored *Mtb* dormant cells (13 months) by the categories in which they can participate.

Table S5 “Consensus” proteins shared between the 3 *Mtb* dormancy models found in the first 200 most abundant. Published data for proteins amount in Loebel and Wayne dormancy models were converted to ranks (places).

Table S6 Overlap between proteins in dormant *M. smegmatis* and *M. tuberculosis* D2 cells.
